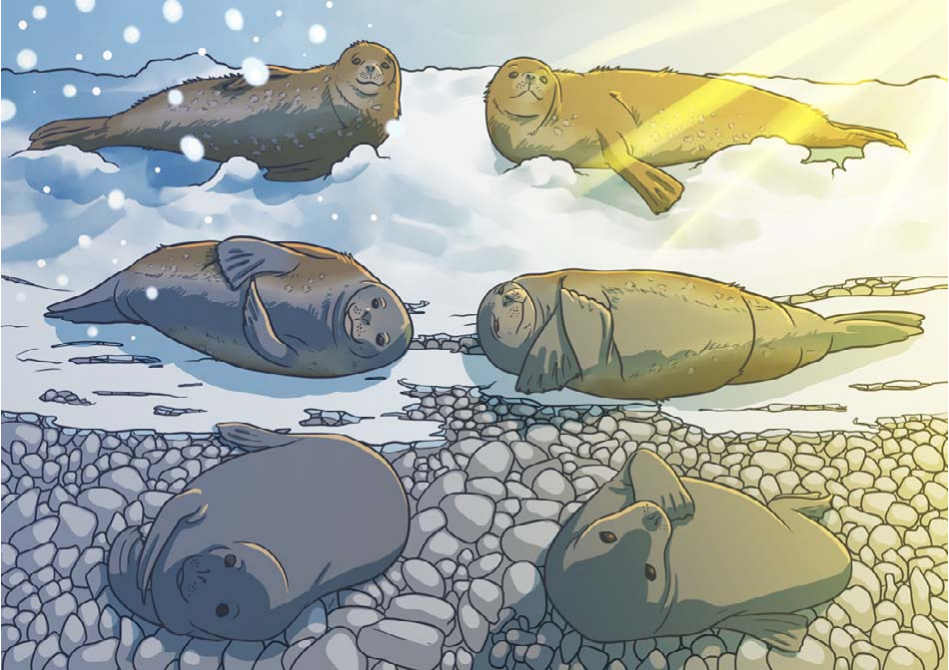# Worried about hair loss? So are these seals

**DOI:** 10.1093/conphys/coaa072

**Published:** 2020-08-25

**Authors:** Lian W Guo

**Affiliations:** Graduate Program in Organismic and Evolutionary Biology, University of Massachusetts, Amherst, MA 01003, USA

Losing and growing hair require a lot of energy! Indeed, a recent study found that annual summer molting in Weddell seals requires a surprising 38% of their resting energy budget. But, molting is not the only energetically expensive task that adult female Weddell seals undergo within the short 7-week window of the Antarctic summer; they must also reproduce and rebuild their blubber stores for winter. If molting requires so much energy, why do Weddell seals do it *every* year?

Fur is an adaptation that insulates seals by trapping a warm layer of air close to skin. Molting replaces old, worn fur with a darker, higher quality fur that maintains efficient insulation. Between losing old fur and growing new fur, seals are temporarily left with little insulation, potentially leading to greater body heat loss to the environment. Weddell seals may then be forced to use more energy to maintain their high body temperature (~37°C), which presents a problem given the competing needs for energy during summer. Do the energetic requirements of molting then make Weddell seal populations more sensitive to changes in food availability or climate?

Skyla Walcott and her colleagues designed a study to understand how environmental factors (e.g. temperature, wind speed) as well as physiology (e.g. molt stage) affect body heat loss and energetic costs. The researchers used infrared cameras and thermocouples to measure surface/core body temperature and heat loss in 93 adult female Weddell seals before and during molting. They used statistical models to determine which factors were most important in affecting body temperature and heat loss.

Walcott’s team found that seals maintained high skin temperatures regardless of molt stage, but seals in early molting lost 25% more heat compared to during the months before molting began. Not surprisingly, fur helped reduce body heat loss by 33%. Heat loss during molting led to a doubling of the energetic costs of thermoregulation (i.e. maintaining their core body temperature). The researchers also observed that lower air temperatures and higher wind speeds increased heat loss and further lowered skin temperatures. These findings suggest that natural selection drove molting to occur during the warm summer months. Higher summer temperatures help minimize heat loss during molt. Summer is also when food is most abundant and shallow in Antarctic waters, making for greater foraging success. These factors allow Weddell seals to recover critical blubber stores depleted by reproduction and supply energy for molting.

Conditions in the Southern Ocean are changing rapidly. While climate change and the resulting warmer temperatures could reduce thermoregulatory costs for Weddell seals, climate change also decreases sea ice habitat and prey availability. With less sea ice, seals may spend more time in the water hunting and have fewer options for hauling out on land during reproduction and molting. If foraging takes longer due to reduced prey availability, it may delay molting into suboptimal cooler fall temperatures. And, we already know that late-molting females show lower success in reproduction, which further emphasizes that the timing of molting is crucial. This information regarding the timing and energy required of molting will help conservationists understand how the changing Antarctic ecosystem affects vulnerable Weddell seal populations.

Illustration by Erin Walsh; Email: ewalsh.sci@gmail.com